# Application of Allele Specific PCR in Identifying Offspring Genotypes of Bi-Allelic *SbeIIb* Mutant Lines in Rice

**DOI:** 10.3390/plants11040524

**Published:** 2022-02-15

**Authors:** Yongqi Jiang, Yinhui Ren, Xin Xu, Hao Wang, Cunxu Wei

**Affiliations:** 1 Key Laboratory of Crop Genetics and Physiology of Jiangsu Province, Jiangsu Key Laboratory of Crop Genomics and Molecular Breeding, Yangzhou University, Yangzhou 225009, China; 182103210@yzu.edu.cn (Y.J.); dx120200177@yzu.edu.cn (Y.R.); mx120211067@yzu.edu.cn (X.X.); mx120211052@yzu.edu.cn (H.W.); 2 Co-Innovation Center for Modern Production Technology of Grain Crops of Jiangsu Province, Joint International Research Laboratory of Agriculture and Agri-Product Safety of the Ministry of Education, Yangzhou University, Yangzhou 225009, China

**Keywords:** clustered regularly interspaced short palindromic repeats (CRISPR)/CRISPR associated (Cas) system, bi-allelic mutation line, allele specific PCR, genotype identification

## Abstract

Bi-allelic mutant lines induced by clustered regularly interspaced short palindromic repeats (CRISPR)/CRISPR associated (Cas) systems are important genetic materials. It is very important to establish a rapid and cheap method in identifying homozygous mutant plants from offspring segregation populations of bi-allelic mutant lines. In this study, the offspring genotypes of rice bi-allelic *starch branching enzyme IIb* mutant lines were identified using the allele specific PCR (AS-PCR) method. The target sequences of two alleles were aligned from their 5′ to 3′ ends, and the first different bases were used as the 3′ ends of mismatch primers. Another mismatched base was introduced at the third nucleotide from the 3′ end of mismatch primer. The PCR reaction mixture and amplification program were optimized according to the differences of mutation target sequence and mismatch primers. The offspring plant genotypes of bi-allelic mutant lines could be accurately identified using the amplified DNA fragments by agarose gel electrophoresis. This study could provide a method reference for the rapid screening of homozygous mutant plants from offspring segregation population of heterozygous and bi-allelic mutant lines.

## 1. Introduction

The studies of gene function and its expression regulation in plants can reveal the molecular mechanism of plant growth and development and the physiological function of resistance to biotic and abiotic stresses through forward and reverse genetics [1,2,3]. The plant mutants may serve as the most important genetic materials to carry out the above studies. The spontaneous mutations are rare and slow in plants. Usually, the mutations of plants are induced easily by physical or chemical mutagens, which can create a large number of genetic mutants and new alleles within a short period for forward genetics [4,5]. However, it is very difficult to screen a desirable phenotype and identify the causal mutations/genes/QTLs through genetic mapping, fine mapping, positional cloning and other advanced genomic approaches. The reverse genetics can reveal the gene function through the mutation or expression regulation of target gene [1,2]. The clustered regularly interspaced short palindromic repeats (CRISPR)/CRISPR associated (Cas) system can easily mutate and edit the target gene, and has been widely used in plant sciences and crop improvement [6,7,8,9,10]. The mutants induced by CRISPR/Cas system usually have many heterozygous and bi-allelic mutant lines, which can produce the homozygous mutant plants in their offspring segregation population [11,12,13,14,15,16,17]. The identification and screening of homozygous mutant plants from segregated populations usually use DNA sequencing, a time-consuming and fund-costing method. Therefore, it is important to establish a rapid and economical method for identifying the offspring plant genotypes of heterozygous and bi-allelic mutant lines.

Allele specific PCR (AS-PCR) can detect the single nucleotide polymorphism (SNP) [18,19,20]. The AS-PCR is simple, rapid, economical, reliable, and does not require high levels of technology and instrument. Therefore, it has been widely used to screen the materials with target SNP genotype from a large number of natural populations [21,22] or mutant segregation populations [15,16,17]. However, it is unclear whether the AS-PCR can detect the offspring genotype of heterozygous and bi-allelic mutant line with non-SNP mutations.

In this study, many bi-allelic mutant lines were detected from rice *starch branching enzyme IIb* (*sbeIIb*) mutant lines induced by CRISPR/Cas9. The mismatch primers were designed according to the bi-allelic mutation sequence, the PCR reaction mixture and amplification program were optimized, and the genotypes of offspring plants of bi-allelic mutant lines were detected. This study could provide an important method reference to detect the offspring plant genotypes of heterozygous and bi-allelic mutant lines and screen the homozygous mutant plants as genetic materials for investing the function and its expression regulation of target gene.

## 2. Results

### 2.1. CRISPR/Cas9 Induced Mutation in SBEIIb Gene and Mutation Types of T1 Lines

**Figure 1:**
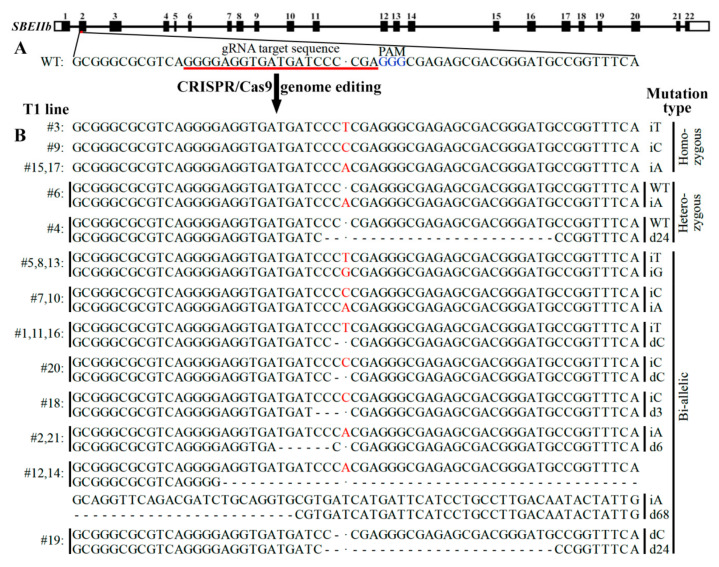
CRISPR/Cas9 induced mutation in *SBEIIb* gene and mutation types of T1 lines. (**A**) A schematic map of the gRNA target sites on the genomic regions of *SBEIIb*. The introns are shown as lines, and the exons are shown as boxes. (**B**) Sequencing results of T1 mutant lines. The inserted nucleotide is highlighted in red, and the dashes indicate deletions.

Twenty-one T1 lines were obtained from rice cultivar Kitaake with *SBEIIb* mutation through CRISPR/Cas9 system. Their leaves were detected for *SBEIIb* target sequences and mutation types through DNA sequencing (Figure 1). Among 21 T1 lines, four lines (#3, 9, 15, and 17) exhibited homozygous mutation with insertion of nucleotide T, C, or A, and two lines had heterozygous mutation with insertion of nucleotide A (#6) or deletion of 24 nucleotides (#4) in mutation chromatid. In total, 15 lines (71.5%) exhibited bi-allelic mutation. Lines (iT/iG) of #5, 8, and 13 had an insertion of nucleotide T in one allele and G in another allele, and lines (iC/iA) of #7 and 10 had an insertion of nucleotide C in one allele and A in another allele. Lines (iT/dC) of #1, 11, and 16 had an insertion of nucleotide T in one allele and a deletion of nucleotide C in another allele, and line (iC/dC) of #20 had an insertion of nucleotide C in one allele and a deletion of nucleotide C in another allele. The deletion of 3 (d3), 6 (d6), 24 (d24), and 68 (d68) nucleotides in one allele were also detected in some lines with an insertion of nucleotide C (iC/d3) (#18), an insertion of nucleotide A (iA/d6) (#2 and 21), a deletion of nucleotide C (dC/d24) (#19), and an insertion of nucleotide A (iA/d68) (#12 and 14) in another allele, respectively (Figure 1). These bi-allelic mutant lines were important genetic materials, and could result in homozygous mutant plants in their offspring segregation population.

### 2.2. Detecting Genotypes of Offspring Segregation Plants from Bi-Allelic Mutant Lines

The bi-allelic mutant lines of #5, 1, 2, and 12 with mutation types of iT/iG, iT/dC, iA/d6, and iA/d68 were used as examples to exhibit whether the AS-PCR method could detect the genotypes of offspring segregation plants from bi-allelic mutant lines. In this study, the mismatched bases were introduced at the first and third nucleotide from the 3′ end of the mismatch forward or reverse primers (Table 1). The melting temperature (Tm) of primer and length of amplified fragment were presented in Table 1.

**Figure 2:**
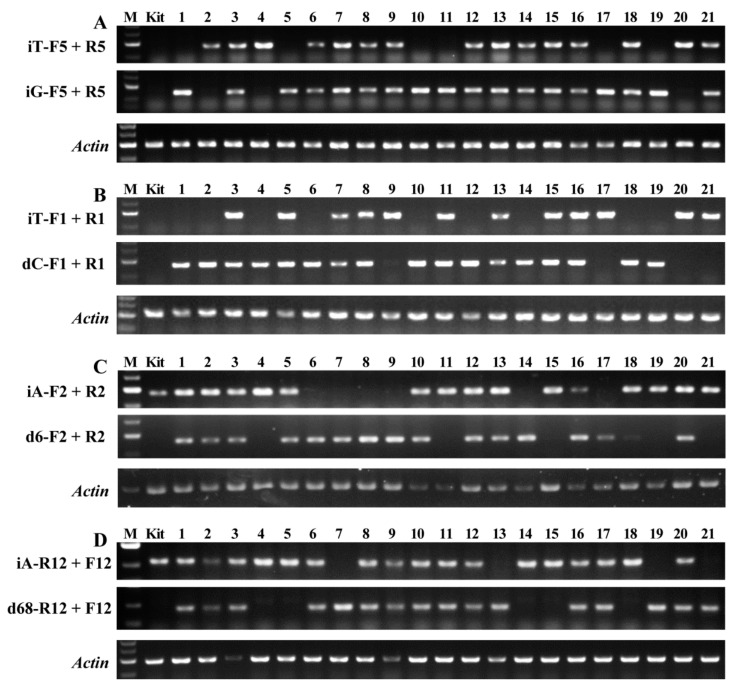
Genotype detection of offspring plants of bi-allelic T1 lines using AS-PCR. (**A**) Bi-allelic mutant line of #5 (iT/iG) with T insertion in one allele and G insertion in another allele; (**B**) bi-allelic mutant line of #1 (iT/dC) with T insertion in one allele and C deletion in another allele; (**C**) bi-allelic mutant line of #2 (iA/d6) with A insertion in one allele and 6 nucleotides deletion in another allele; and (**D**) bi-allelic mutant line of #12 (iA/d68) with A insertion in one allele and 68 nucleotides deletion in another allele. M, DNA marker; Kit, wild type rice Kitaake; and 1–21, partial plants of T2 segregation population. The primers are shown in Table 1.

It is very important to optimize the reaction system of AS-PCR. Compared with routine PCR, the reaction mixture and PCR amplification program were optimized according to the mutation sequences and mismatch primers (Table 2, Figure 2). In 10 μL reaction volume, the 0.5 μL primer (10 μM) and 1.5 μL template DNA (150 ng/μL) were suitable for mutant lines of #5 and 12, and 0.3 μL primer (10 μM) and 1.0 μL template DNA (150 ng/μL) were suitable for mutant lines of #1 and 2 (Table 2). In the 28 PCR cycles including denaturation, annealing and extension, the annealing temperature 58 °C was suitable for mutant line of #5 and 1, 57 °C for mutant line of #2, and 53 °C for mutant line of #12 (Table 2). The plant genotypes could be detected clearly in T2 segregation populations of bi-allelic mutant lines (Figure 2). For example, the offspring plants of bi-allelic line of #5 had homozygous mutation genotype with insertion of nucleotide T (iT) for plant of #2, 4, and 20 and nucleotide G (iG) for plant of #1, 5, 10, 11, 17, and 19 and bi-allelic genotype (iT/iG) for plant of #3, 6–9, 12–16, 18, and 21 (Figure 2A). The segregation of offspring genotypes in bi-allelic mutation lines was summarized in Table 3. All the studied bi-allelic mutant lines were in line with the Mendelian segregation ratio (1:2:1) in their offspring plants.

### 2.3. Accuracy of Genotype Detected by AS-PCR

**Figure 3:**
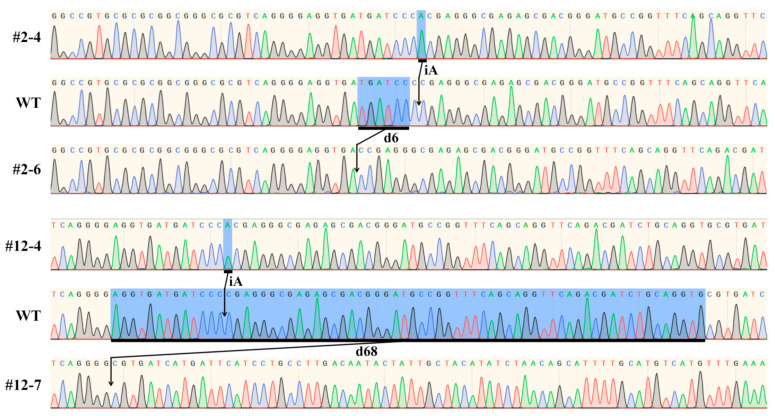
Sequencing analysis of homozygous offspring plant of #4 and 6 in bi-allelic mutant line of #2 (Figure 2C) and #4 and 7 in bi-allelic mutant line of #12 (Figure 2D).

In order to confirm the accuracy of the genotype detected by AS-PCR, the *SBEIIb* target DNA of the homozygous offspring plant of #4 and 6 in bi-allelic mutant line of #2 (Figure 2C) and #4 and 7 in bi-allelic mutant line of #12 (Figure 2D) was sequenced. The sequencing results exhibited the homozygous mutation with the insertion of A and deletion of six nucleotides in the offspring plant of #4 and 6 in the bi-allelic mutant line of #2, respectively. The homozygous mutations with the insertion of A and deletion of 68 nucleotides were also detected in offspring plant of #4 and 7 in bi-allelic mutant line of #12, respectively (Figure 3). The sequencing results confirmed the accuracy of offspring plant genotypes in bi-allelic mutant lines detected by the AS-PCR method.

**Figure 4:**
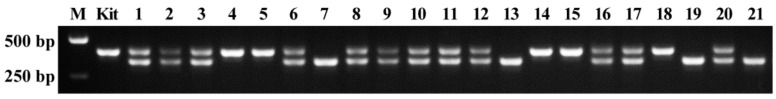
Genotype detection of offspring plants of iA/d68 T1 line using routine PCR. M, DNA marker; Kit, wild type rice Kitaake; and 1–21, the same plants of T2 segregation population detected in Figure 2D.

The mutation *SBEIIb* target sequences of offspring plants in the bi-allelic mutant line of #12 (Figure 2D) were also amplified using routine PCR primers and system. The homozygous mutation plants with an insertion of A and deletion of 68 nucleotides all exhibited one band with large molecular weight for the former and small molecular weight for the latter in the agarose gel, and the bi-allelic mutation plants had two bands (Figure 4). The genotypes of offspring plants in bi-allelic mutant line of #12 detected by routine PCR agreed with those detected by AS-PCR (Figure 2D and Figure 4), indicating that the offspring genotypes of bi-allelic mutant lines could be detected accurately by AS-PCR method.

## 3. Discussion

Plant mutants are important genetic materials in revealing gene function and its regulation, and providing germplasm resources for crop breeding. The physical mutation, chemical mutation, and T-DNA insertion mutation are conventional mutation methods. However, their mutation ratios are low, and the mutation sites are random and unknown. The CRISPR/Cas9 system can easily produce target gene mutation, and has been widely used in plant mutant creation, especially for reverse genetics in revealing target gene function and regulation [6,7,8,9,10]. The Cas9 protein functions as a nuclease and is guided to the target site by the engineered single guide RNA (gRNA) including 20 specific nucleotides of the selected gene to determine the site-specific targeting. The double strand breaks of DNA are caused by precise cleavage of the Cas9 protein at the target site in the genome, which can be then repaired by non-homologous end-joining (NHEJ) or homologous recombination (HR). The NHEJ is error-prone and often introduces base substitution, insertion, or deletion at the cleavage site, producing potential genetic materials for research [11,23]. The base substitution can result in the substitution of amino acids when the targeted gene encodes a protein. In addition, the insertion and deletion of bases can not only generate frame-shifted knockout mutations of targeted protein, but also produce protein with the addition or deficiency of several amino acids when the base number of insertion and deletion is a multiple of 3 [12,13]. Though the mutation ratio of the CRISPR/Cas9 system is high, many heterozygous and bi-allelic mutant lines are produced [11,12,13,14,15,16,17]. Therefore, it is important to identify the offspring plant genotypes of heterozygous and bi-allelic mutant lines to screen the homozygous mutant plants.

The AS-PCR can detect the SNP genotypes, but needs three primers containing one common primer and two specific mismatch primers. The common primer is designed in the conventional way of routine PCR, but the two mismatch primers are designed according to SNP sites, and their 3′ ends are identical to the base of SNP site, respectively. In addition, it’s necessary to artificially introduce a mismatched base near the 3′ end of the mismatch primer to produce the difference of amplification detected by agarose gel electrophoresis. The mismatch primer has an amplification product in agarose gel when it has one mismatched base in one genotype, but no amplification product in another genotype due to having two mismatched bases [18,19,20]. The AS-PCR has been widely used in identifying SNP genotype, especially in rapidly screening different genotype plants from natural population and a large amount of segregated population in crop molecular assistant breeding [15,16,17,22,24]. However, it is unclear whether the AS-PCR can be used to detect the offspring plant genotypes of heterozygous and bi-allelic mutant lines, especially for base insertion or deletion. The present study indicated that the AS-PCR could rapidly and accurately detect the homozygous and heterozygous mutant plants with base substitution, insertion, and detection from a large number of offspring segregation populations of heterozygous and bi-allelic mutant lines induced by CRISPR/Cas9 system. The homozygous genotype was detected only using the mismatch primer with one mismatched base, but not detected using another mismatch primer with two mismatched bases, and the heterozygous genotype was detected using the two mismatch primers (Figure 2 and Figure 3).

For AS-PCR, the position of the introduced mismatched base is very important. Some of the literature introduces the mismatched base at the penultimate (−2) or antepenultimate (−3) position of the 3′ end of mismatch primer to improve the specificity of detection and avoid false positives. Compared with the −2 and −4 positions, the introduction of a mismatched base at the −3 position not only ensures high specificity, but also avoids low yield of the target amplification product, indicating that the mismatched base position at −3 is the best [25,26]. In the present study, we introduced the mismatched base at the −3 position of the 3′ end of mismatch primers. It has been reported that the type of mismatched bases also affect the specificity of primers [24,27,28,29,30]. The specific mismatch primers in this study were designed according to Little’s original principle (Table 1, [24]). In addition, when the Tm value or GC content of the forward mismatch primers are not suitable for the target sequence, the reverse mismatch primers can be tried, such as the primers of iA-R12 and d68-R12 for #12 (Table 1). In general, the primer concentration, amount of Taq DNA polymerase and template DNA in the reaction system of AS-PCR are much less than those of routine PCR. The annealing temperature is also one of the important factors affecting the amplification efficiency. In this study, we reduced the amount of the above components in the reaction mixture and the number of amplification cycles, and selected the appropriate annealing temperature for each group of primers to avoid non-specific amplification (Table 1 and Table 2).

The DNA sequencing is often used to detect the genotype of a large number of offspring plants of heterozygous and bi-allelic mutant lines [13]. This method exhibits high accuracy, but it is fund-costing and time-consuming. In this study, the accuracy of AS-PCR was confirmed by DNA sequencing (Figure 3), indicating that the AS-PCR is a simple, rapid, cheap, and accurate method for detecting the offspring genotypes of heterozygous and bi-allelic mutant lines. Some references develop a tetra-primer amplification refractory mutation system PCR (tetra-primer ARMS PCR) from AS-PCR to detect SNP genotypes, which needs two common outer primers and two mismatch inner primers in only one PCR reaction to distinguish homozygotes and heterozygotes and their genotypes [27,31]. Though the tetra-primer ARMS PCR has more efficiency than the AS-PCR, it requires very high primer specificity, amplification system, and cycling conditions. In order to ensure the accuracy of detection, the optimal reaction conditions including the concentration of each primer, annealing temperature, and reaction cycles must be tried many times before detection [27,31]. It is more simple and easier to optimize the reaction condition for AS-PCR than the tetra-primer ARMS PCR. The AS-PCR has been widely used to detect the SNP genotypes in many plant species, such as rice, barley, rapeseed, sesame, oleracea, and persimmon [15,16,17,18,19,20,21,22,32,33]. Based on the uniformity of designing mismatch primers, the AS-PCR can be used to rapidly and accurately identify the offspring genotypes of bi-allelic and heterozygous mutant lines with base substitution, insertion and deletion in not only *SBEIIb* of rice but also other plant species and genes.

## 4. Materials and Methods

### 4.1. Plant Materials

**Figure 5:**
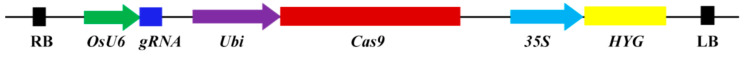
The vector construction diagram.

The *japonica* rice variety Kitaake is regarded as a model rice variety due to its short life cycle (9 weeks) and photoperiod insensitivity [34]. In this study, the *SBEIIb* gene of Kitaake was edited using CRISPR/Cas9 system by Hangzhou Biogle Co., LTD. (Hangzhou, China). As shown in Figure 1A, the designed target sequence GGGGAGGTGATGATCCCCGA is on the second exon of *SBEIIb* gene. The GGG next to the target site is protospacer adjacent motif (PAM). The vector used by the company is BGK032, which contains a hygromycin resistance gene (*HYG*) for callus selection. The *Cas9*, *HYG,* and *gRNA* on the backbone are driven by maize *Ubi* promoter, CaMV *35S* promoter, and rice *U6* promoter, respectively (Figure 5). The constructed vector was transferred into *Agrobacterium tumefaciens* strain EHA105 and then transformed into callus induced from mature seeds. The induced *sbeIIb* T1 mutant lines and their T2 offspring plants were planted in the transgenic closed experimental field of Yangzhou University (Yangzhou, China) under conventional cultivation and management condition. Leaves and mature grains were harvested from single plants.

### 4.2. Leaf DNA Extraction

Genomic DNA was extracted from leaves of a single plant using the CTAB method. Briefly, leaf pieces were ground into powder in the 2-mL centrifuge tube and incubated in 400 μL 2% CTAB extraction solution (100 mM Tris-HCl, pH 8.0, 2% CTAB, 20 mM EDTA, 1.4 M NaCl) with intermittent shaking at 65 °C for 40 min. The sample was added 400 μL chloroform-isoamyl alcohol (24:1), mixed fully by inverting the tube, and stood at room temperature for 15 min before centrifugation (12,000× *g*, 5 min). The supernatant was transferred to a new 1.5-mL centrifuge tube carefully, added twice volume of ethanol, mixed fully by inverting the tube, and stood at −20 °C for over 30 min before centrifugation (12,000× *g*, 5 min). To the precipitate, 0.5 mL 70% ethanol was added, and centrifuged (12,000× *g*, 5 min) after 5 min at room temperature. The above wash was repeated again. The sample was air-dried, dissolved in 50 μL sterilized deionized water, and stored at −20 °C. The DNA concentration was adjusted to 150 ng/µL with sterilized deionized water before use.

### 4.3. Routine PCR and DNA Sequencing

The target DNA fragment was amplified using routine PCR method. The reaction mixture (50 µL) contained 25 µL 2 × Rapid Taq Master Mix (P222, Vazyme, Nanjing, China), 2.0 µL forward primer (10 µM), 2.0 µL reverse primer (10 µM), 1.5 µL template DNA, and 19.5 µL sterilized deionized water. The primers were presented in Table 1. The PCR program was set as initial denaturation at 95 °C for 5 min, 31 cycles of denaturation at 95 °C for 30 s, annealing at 56 °C for 30 s and extension 72 °C for 3 s, final extension at 72 °C for 7 min, and storage at 16 °C. Finally, the amplified product (5 µL) was taken and detected by electrophoresis with 1% agarose gel at 120 V, 20 min, and the rest was sent to Tsingke Biotechnology (Nanjing, China) for sequencing.

### 4.4. Allele-Specific Primer Design

The allele-specific mismatch primers (Table 1) were designed according to the target mutation sequences with reference to Little’s method [24]. Briefly, the target sequences of two alleles were aligned from their 5′ to 3′ ends, and the first different bases were used as the 3′ ends (−1 position) of mismatch primers. Another mismatched base was introduced at the third nucleotide (−3 position) from the 3′ end of mismatch primer, and followed the below principle. When the first (−1 position) mismatched base exhibited a strong mismatch of G/A, C/T, or T/T between two alleles, the second (−3 position) mismatched base was introduced as a weak mismatch of C/A or G/T between mismatch primer and parental sequence, and vice versa. When the first mismatched base exhibited a medium mismatch of A/A, C/C, or G/G between two alleles, the second mismatched base was also introduced as a medium mismatch of A/A, C/C, or G/G between mismatch primer and parental sequence. The primer design of iT-F1 and dC-F1 was explained as an example as below. After aligning the target sequences of two alleles of #1 line from 5′ to 3′ end, the first different bases were T and G (Figure 1B), resulting in that the last base (−1 position) at the 3′ end of primer was T and G for iT-F1 and dC-F1, respectively. The C at the third (−3) position of the 3′ end of primers was replaced with T to introduce the strong C/T mismatch because the T/G mismatch at −1 position was a weak mismatch. Another common primer was designed using conventional method of routine PCR. In addition, the good primers need to meet the essential conditions that their lengths were 18–25 bp, Tm values were as close as possible, and amplified product length was 150–600 bp.

### 4.5. AS-PCR and Genotype Detection

For AS-PCR, both reaction mixture and PCR program including concentration of primers and template DNA, reaction cycle, and annealing temperature were optimized according to the mutation sequences and mismatch primers (Table 2). The amplified DNA could be detected by electrophoresis with 1% agarose gel when the target DNA sequence had a mismatched base with the primer, but could not be detected when it had two mismatched bases with the primer.

### 4.6. Detection of Genotype with Large Fragment Deletion

For the mutant line with a deletion of 68 nucleotides, the common primers were designed to amplify the short DNA fragments containing the mutation site (Table 1). The reaction mixture (10 µL) contained 5 µL 2 × Taq Master Mix (Quick Load) (E005-02A, Novoprotein Scientific Inc., Suzhou, China), 0.75 µL forward primer (10 µM), 0.75 µL reverse primer (10 µM), 1.0 µL template DNA, and 2.5 µL sterilized deionized water. The PCR program was set as initial denaturation at 95 °C for 5 min, 32 cycles of denaturation at 95 °C for 30 s, annealing at 56 °C for 30 s and extension at 72 °C for 28 s, final extension at 72 °C for 7 min, and storage at 16 °C. Finally, the amplified products (6 µL) were detected by electrophoresis with 2% agarose gel at 75 V for 40 min to visibly separate the two DNA bands with different molecular weights.

### 4.7. Statistical Analysis

The data were analyzed using SPSS 19.0 Statistical Software Program (IBM Company, Chicago, IL, USA).

## 5. Conclusions

In this study, some rice bi-allelic mutant lines with base substitution, insertion, and deletion induced by CRISPR/Cas9 were used as plant materials to verify whether the AS-PCR could detect the offspring genotypes of heterozygous and bi-allelic mutant lines with non-SNP mutation. The specific mismatch primers were designed according to different mutation sites, where the first different base between two alleles aligned from 5′ to 3′ end was designed as the 3′ end (−1 position) of mismatch primers, and a mismatched base was introduced into the −3 position of the primers. The specificity of amplification was detected by agarose gel electrophoresis after PCR reaction mixture including primer and template DNA contents and amplification program including reaction cycle and annealing temperature were optimized. Three offspring genotypes of the bi-allelic mutant line could be identified easily by AS-PCR, and the detection accuracy was confirmed by the DNA sequencing and the routine PCR with common primers for the mutation with large fragment deletion. In conclusion, the AS-PCR is a simple, rapid, cheap, and accurate method for detecting the offspring genotypes of heterozygous and bi-allelic mutant lines with base substitution, insertion, and detection.

## Figures and Tables

**Table 1:** Primers for detecting offspring plant genotypes of bi-allelic mutant lines.

Rice Materials	Primer	Tm (°C)	Amplified Fragment Length (bp)
All lines	F ^a^: 5′-CGTGAGGAGGGTTTAGGTGGAA-3′	59.5	680
	R ^a^: 5′-TCCCGTCACAAACAGAAATCAA-3′	53.9	
#5 (iT/iG)	iT-F5 ^b^: 5′-CAGGGGAGGTGATGATCTCT-3′	57.5	424
	iG-F5 ^b^: 5′-CAGGGGAGGTGATGATCTCG-3′	59.5	424
	R5: 5′-ACAACTGAACGGAATGAGCG-3′	55.4	
#1 (iT/dC)	iT-F1 ^b^: 5′-CAGGGGAGGTGATGATCTCT-3′	57.5	424
	dC-F1 ^b^: 5′-CAGGGGAGGTGATGATCTCG-3′	59.5	422
	R1: 5′-ACAACTGAACGGAATGAGCG-3′	55.4	
#2 (iA/d6)	iA-F2 ^b^: 5′-CGCGTCAGGGGAGGTTAT-3′	57.2	429
	d6-F2 ^b^: 5′-CGCGTCAGGGGAGGTTAC-3′	59.5	422
	R2: 5′-ACAACTGAACGGAATGAGCG-3′	55.4	
#12 (iA/d68)	iA-R12 ^b^: 5′-AGGATGAATCATGATCACCCA-3′	53.7	292
	d68-R12 ^b^: 5′-AGGATGAATCATGATCACCCC-3′	55.6	223
	F12: 5′-GCCACCTTGTTGTTCTCGTC-3′	57.5	
#12 (iA/d68)	F ^c^: 5′-GCCACCTTGTTGTTCTCGTC-3′	57.5	413/344
	R ^c^: 5′-AGACGTGGACTGCGTGAAAT-3′	55.4	

^a^ The primer is designed for routine PCR and DNA sequencing in all rice materials, and the amplified fragment length of 680 bp is for wild type rice. ^b^ The mismatch primer is designed for AS-PCR, and the underlined base in the primer is additional mismatch nucleotide. ^c^ The primer is designed for routine PCR to amplify DNA fragment of offspring plants in bi-allelic line #12, and the amplified fragment length of 413 and 344 bp is for genotype with insertion of A and deletion of 68 bases, respectively.

**Table 2:** The reaction system of AS-PCR for detecting offspring genotypes in bi-allelic mutant lines.

PCR Reaction System	#5	#1	#2	#12
Reaction mixture (10 μL)				
2 × Taq Master Mix (Quick Load) ^a^	2.5 μL	2.5 μL	2.5 μL	2.5 μL
Forward primer (10 μM)	0.5 μL	0.3 μL	0.3 μL	0.5 μL
Reverse primer (10 μM)	0.5 μL	0.3 μL	0.3 μL	0.5 μL
Template DNA (150 ng/μL)	1.5 μL	1.0 μL	1.0 μL	1.5 μL
Sterilized deionized water	5 μL	5.9 μL	5.9 μL	5 μL
PCR program				
Stage 1: Initial denaturation (5 min)	95 °C	95 °C	95 °C	95 °C
Stage 2: 28 reaction cycles: Denaturation (30 s)	95 °C	95 °C	95 °C	95 °C
Annealing (30 s)	58 °C	58 °C	57 °C	53 °C
Extension (28 s)	72 °C	72 °C	72 °C	72 °C
Stage 3: Final extension (7 min)	72 °C	72 °C	72 °C	72 °C
Stage 4: Storage (forever)	16 °C	16 °C	16 °C	16 °C

^a^ E005-02A, Novoprotein Scientific Inc., Suzhou, China.

**Table 3:** The segregation and Chi-square of offspring genotypes in bi-allelic mutant lines.

T1 Line	Genotype	Segregation of T2 Plants			
		Total Number	Targeted Mutation Number	Expected Segregation Ratio	χ^2^
#5	iT/iG	33	6 iT, 18 iT/iG, 9 iG	1:2:1	0.818 ^NS^
#1	iT/dC	53	14 iT, 22 iT/dC, 17 dC	1:2:1	1.867 ^NS^
#2	iA/d6	29	7 iA, 15 iA/d6, 7 d6	1:2:1	0.035 ^NS^
#12	iA/d68	30	8 iA, 14 iA/d68, 8 d68	1:2:1	0.133 ^NS^

^NS^ No significant deviation.

## Data Availability

The data presented in this study are available on request from the corresponding authors. The data are not publicly available due to ongoing research.
